# Alpha-Synuclein as a Biomarker of Parkinson’s Disease: Good, but Not Good Enough

**DOI:** 10.3389/fnagi.2021.702639

**Published:** 2021-07-08

**Authors:** Upasana Ganguly, Sukhpal Singh, Soumya Pal, Suvarna Prasad, Bimal K. Agrawal, Reena V. Saini, Sasanka Chakrabarti

**Affiliations:** ^1^Department of Biochemistry and Central Research Laboratory, Maharishi Markandeshwar Institute of Medical Sciences and Research, Maharishi Markandeshwar Deemed University, Ambala, India; ^2^Department of General Medicine, Maharishi Markandeshwar Institute of Medical Sciences and Research, Maharishi Markandeshwar Deemed University, Ambala, India; ^3^Department of Biotechnology, Maharishi Markandeshwar Engineering College, Maharishi Markandeshwar Deemed University, Ambala, India

**Keywords:** imaging biomarkers, cerebrospinal fluid, α-synuclein oligomers, phosphorylated α-synuclein, extracellular vesicles, metabolomics, brain-derived neurotrophic factor, uric acid

## Abstract

Parkinson’s disease (PD) is the second most common neurodegenerative disorder of the elderly, presenting primarily with symptoms of motor impairment. The disease is diagnosed most commonly by clinical examination with a great degree of accuracy in specialized centers. However, in some cases, non-classical presentations occur when it may be difficult to distinguish the disease from other types of degenerative or non-degenerative movement disorders with overlapping symptoms. The diagnostic difficulty may also arise in patients at the early stage of PD. Thus, a biomarker could help clinicians circumvent such problems and help them monitor the improvement in disease pathology during anti-parkinsonian drug trials. This review first provides a brief overview of PD, emphasizing, in the process, the important role of α-synuclein in the pathogenesis of the disease. Various attempts made by the researchers to develop imaging, genetic, and various biochemical biomarkers for PD are then briefly reviewed to point out the absence of a definitive biomarker for this disorder. In view of the overwhelming importance of α-synuclein in the pathogenesis, a detailed analysis is then made of various studies to establish the biomarker potential of this protein in PD; these studies measured total α-synuclein, oligomeric, and post-translationally modified forms of α-synuclein in cerebrospinal fluid, blood (plasma, serum, erythrocytes, and circulating neuron-specific extracellular vesicles) and saliva in combination with certain other proteins. Multiple studies also examined the accumulation of α-synuclein in various forms in PD in the neural elements in the gut, submandibular glands, skin, and the retina. The measurements of the levels of certain forms of α-synuclein in some of these body fluids or their components or peripheral tissues hold a significant promise in establishing α-synuclein as a definitive biomarker for PD. However, many methodological issues related to detection and quantification of α-synuclein have to be resolved, and larger cross-sectional and follow-up studies with controls and patients of PD, parkinsonian disorders, and non-parkinsonian movement disorders are to be undertaken.

## Introduction

Parkinson’s disease (PD) is a complex and progressive neurodegenerative disorder that mainly affects the elderly population and appears sporadically and in familial forms. Familial type accounts for only 5–10% of PD patients, while the vast majority of PD subjects suffer from the sporadic form, which has a multi-factorial origin (Kalia and Lang, 2015). The cardinal signs of PD are motor impairments such as resting tremor, bradykinesia, muscular rigidity, postural instability (Jankovic, 2008; Greenland and Barker, 2018; Kouli et al., 2018). Flexed posture and ‘freezing’ are also characteristic of PD, and many secondary motor symptoms occur during the disease (Jankovic, 2008). Non-motor symptoms like autonomic dysfunction, anosmia, sleep disorders, hallucinations, depression, and dementia also appear; some non-motor symptoms appear even before the advent of the motor symptoms (Jankovic, 2008; Greenland and Barker, 2018). However, the term ‘parkinsonian disorders’ encompasses other diseases like multiple system atrophy (MSA), progressive supranuclear palsy, corticobasal degeneration, drug-induced parkinsonism, and post-encephalitic parkinsonism, with similar or overlapping clinical features as in PD (Dickson, 2012). The diagnosis of PD is essentially clinical, but when the presentation is not classical, PD may be confused with other parkinsonian disorders or several other movement disorders. Thus, a PD- specific biomarker would be useful for differential diagnosis in cases of non-classical presentation. Further, the motor symptoms of PD develop due to the dopaminergic neuronal loss of substantia nigra of the mid-brain, and there is already massive dopaminergic neurodegeneration by the time the patient presents clinically to the physician. A neuroprotective therapy initiated early would be an ideal treatment option for PD, but a clinically useful neuroprotective drug for PD is not available so far. Identifying and validating a putative neuroprotective drug through randomized controlled trials would be difficult unless PD can be identified unequivocally at a very early stage. Therefore, a conveniently detectable biomarker for early-stage diagnosis of PD will serve a very important purpose for clinical trials of neuroprotective compounds and early initiation of neuroprotective therapy.

Although the term ‘biomarker’ is often used in a vast sense, we will restrict the term in this review as a tool to aid in the diagnosis of the disease or its prognostic evaluation. The biomarker for PD is often categorized as genetic, imaging-based, or biochemical biomarkers (Emamzadeh and Surguchov, 2018; He et al., 2018). However, the genetic biomarker is especially useful for the familial form of PD, which accounts for a small subset of PD subjects usually presenting as early-onset cases with a positive family history. For most other cases of late-onset sporadic PD, an imaging-based or a biochemical biomarker would be the ideal choice. In this review, we would present an overview of epidemiology, diagnosis, pathogenesis, and pathology of the disease and briefly discuss the genetic and other biomarkers before going into a detailed analysis of α-synuclein as a candidate biomarker for PD. Ideally, a biomarker should be related to the pathogenesis of the disease, and α-synuclein meets this criterion very aptly in the context of PD. However, for a disease like PD affecting the basal ganglia and other discrete regions of the brain primarily, the changes in the level of α-synuclein may be manifested consistently only in cerebrospinal fluid (CSF), and this may impede its use as a promising biomarker for PD. Thus, it will be interesting to review the available studies measuring the levels of α-synuclein in peripheral circulation or other accessible body fluids in PD. Further, it will be essential to scrutinize the existing literature for various limitations of different studies on the biomarker potential of α-synuclein.

## PD: An Overview

### Epidemiology

Parkinson’s disease is the second most common neurodegenerative disease of the elderly population, whose prevalence is 0.5 to 1% in the age group of 65–69 years, and it gradually rises with the increasing age (Kouli et al., 2018). The global burden of PD increased from 2.5 million in 1990 to 5.1 in 2016, which is variably distributed in different countries, and the rise in PD population over time cannot be solely attributed to the increase in the aged population in different countries during this period (GBD 2016 Neurology Collaborators, 2018). Many studies have reported variable prevalence of PD according to gender and in different countries and age groups. A meta-analysis of PD prevalence reported in 2014 that the prevalence increases with age and is significantly higher in males in the age group of 50 to 59 years (Pringsheim et al., 2014). This meta-analysis further showed that in the age group of 70 to 79 years, a higher prevalence of PD is noted in Europe, North America, and Australia than in Asia. A detailed and recent meta-estimate of PD prevalence collected from different sources in several regions of North America found the prevalence of 572 per 1,00,000 above the age of 45 years (Marras et al., 2018). This study analyzed in detail the reasons for the variable prevalence of PD reported in earlier studies. As observed in this study, the prevalence was more in the males, increased with age, and showed some regional variations. In a study based in Korea, age and gender standardized prevalence of PD in 2015 was estimated to be 139.8 per 1,00,000 with females having a higher prevalence, and both the incidence and prevalence of PD increased continuously from 2010 to 2015 in this country (Park et al., 2019). In a comparative analysis of different descriptive studies and meta-analyses on the prevalence of PD in eastern and western nations, Abbas et al. (2018) showed an increased prevalence with age, male predominance, and interestingly a lower prevalence of this disorder in the eastern nations. The differences in the prevalence of PD in different countries could be related to inappropriate sample selections, limitations of various data collection methods and analyses, and different life expectancies in different populations. Alternatively, this difference may arise from the variances in genetic or environmental risk factors for PD present in different populations.

### Diagnosis of PD

When PD presents with typical motor symptoms as described above, the diagnosis of the disease can be made with a great degree of certainty. The presence of autonomic dysfunction, some sensory loss like anosmia, ageusia, sleep disorders, unilateral onset and persistent asymmetry of symptoms, positive response to dopaminergic therapy, and levodopa-induced dyskinesia can strengthen the diagnosis (Jankovic, 2008; Greenland and Barker, 2018; Marsili et al., 2018). In many cases, the disease may be confused with other ‘parkinsonian disorders,’ where a careful recording of history, e.g., exposure to pesticides, encephalitis, certain antipsychotic drugs, or the presence of certain exclusion criteria or brain imaging studies may help to clinch the diagnosis. Since the confirmation of PD can be established only by post-mortem histopathology, many diagnostic criteria have been proposed by expert groups (Jankovic, 2008; Marsili et al., 2018). The severity of symptoms, associated disability, and the progress of the disease can be assessed by using several rating scales. Hoehn and Yahr (H-Y) scale was the oldest one which divides PD into several stages based on the presence or absence of a battery of clinical symptoms indicative of motor impairment and disability. Many other rating scales have since been utilized to obtain a global estimate of disease severity or specific aspects of the disease. The unified Parkinson’s disease rating scale (UPDRS), later upgraded by the expert group of Movement Disorder Society (MDS-UPDRS) in 2008, takes into consideration of motor disability, motor complications, activities of daily living, non-motor symptoms, including changes in mood, behavior, and intellectual functions (Martínez-Martín et al., 2015). Other global assessment methods like the clinical impression of severity index for Parkinson’s disease (CISI-PD), Parkinson’s disease composite scale (PDCS), and several other forms of assessment of specific PD-related symptoms are also in use (Martínez-Martín et al., 2015, 2016; Pintér et al., 2019). The importance of these rating scales and their correlation with each other are important areas of clinical PD research (Martínez-Martín et al., 2016; Ayala et al., 2017; Skorvanek et al., 2017).

The familial forms of PD often have an earlier onset and sometimes an aggressive progression than sporadic forms. Still, clinical features are very similar to that of sporadic type in most cases. The differential diagnosis of PD includes not only ‘parkinsonian disorders,’ but also several other disorders like essential tremor, dystonic tremor, neurodegeneration with brain iron accumulation (NBIA), Huntington’s disease, Wilson’s disease, spinocerebellar ataxia, and idiopathic basal ganglia calcification (Greenland and Barker, 2018).

### Pathology and Pathogenesis

The post-mortem macroscopic examination of a PD brain shows characteristically a loss of pigmented region in the substantia nigra of the mid-brain, which microscopically corresponds to a loss of neuromelanin-containing dopaminergic neurons of pars compacta of substantia nigra (Hartmann, 2004; Dickson, 2012). The neuronal loss in the PD brain is not restricted to substantia nigra only, but catecholaminergic as well as non-catecholaminergic neurons in the dorsal motor nucleus of the vagus, olfactory bulb, ventral tegmental region, locus coeruleus, raphe nucleus, and nucleus basalis of Meynert, and the neuronal loss is associated with reactive gliosis (Alexander, 2004; Hartmann, 2004; Dickson, 2012; Giguère et al., 2018). Another characteristic feature of PD pathology is the presence of eosinophilic inclusions (5–30 μ in diameter) in the soma of the surviving neurons (Lewy bodies or LBs) which are visible in routine histopathological staining (Hartmann, 2004; Dickson, 2012; Kouli et al., 2018). The LBs can be immunostained by using antibodies against α-synuclein, and such immuno-staining for α-synuclein also reveals the thread-like inclusion structures in the neuronal processes called Lewy neurites (LNs) (Hartmann, 2004; Dickson, 2012; Kouli et al., 2018). Although α-synuclein is the predominant protein in LBs and LNs, other proteins like ubiquitin, neurofilament protein, ubiquitin-binding protein p62, tubulin, and synphilin-1 also occur in such structures. Somewhat pale staining structures with an ill-defined outline, immuno-reactive to α-synuclein, which are presumably the early stages of LBs, are also seen within neurons in substantia nigra as well as other parts of the PD brain including cortex and amygdala (Braak et al., 2004; Hartmann, 2004; Dickson, 2012; Kouli et al., 2018). From extensive studies with post-mortem PD brains, Braak et al. (2004) have proposed a Lewy-pathology-based staging (1 to 6) of PD. According to their study, the disease begins (Stage 1and 2) in the anterior olfactory nucleus and dorsal vagal nuclei and spreads predictably to reach the locus coeruleus in the pons, substantia nigra, and other nuclei in the mid-brain and fore-brain (Stage 3 and 4) and finally in the different neocortical areas. However, other studies have contradicted this staging and propagation of PD pathology (Dickson, 2012; Jellinger, 2019). One characteristic feature of PD pathology has been the selective vulnerability of specific neurons to develop LBs and LNs and subsequent death while sparing others in close anatomical contact with degenerating neurons. Recent experimental studies further suggest that α-synuclein pathology might begin in the periphery, and the pathological form of the protein may be transferred via the vagal nerve by retrograde transport from the gut wall to the dorsal nucleus of the vagus in the brain, which was originally hypothesized by Braak et al. (2004; Holmqvist et al., 2014). The pathogenic mechanisms of PD have been explored in post-mortem PD brains and experimental models, including PD models in transgenic organisms, toxin-induced neurodegeneration in cultured catecholaminergic cell lines, and various toxin-based animal models of PD. Identifying genes responsible for familial PD and examining the mechanisms of actions of their protein products like α-synuclein, PARKIN, PINK1, LRRK2, and DJ-1 in model systems have provided important clues in understanding the PD pathogenesis both in the familial and sporadic forms. Similarly, exposure to pesticides like paraquat or other types of toxin like MPTP has been implicated in the genesis of PD, resulting in the development of several toxin-based animal models. In general, such studies indicate that oxidative stress, mitochondrial dysfunction, metal accumulation, proteinopathy, and inflammatory reactions are some of the interdependent mechanisms that contribute to neurodegeneration in PD (Jenner, 2003; Eriksen et al., 2005; Schapira, 2007; Dawson et al., 2010; Jackson-Lewis et al., 2012; Gubellini and Kachidian, 2015; Wrangel et al., 2015; Ganguly et al., 2017). The details of PD pathogenesis are not within the purview of this review, and no coherent picture has so far emerged to define the neurodegenerative processes in PD clearly. However, it will not be out of place if we present a brief update of important elements of PD pathogenesis before analyzing the role of α-synucleinopathy in this process.

#### Mitochondrial Dysfunction in PD

Mitochondrial complex I inhibition in substantia nigra in post-mortem PD brain was an early finding which was later reported in platelets and skeletal muscles of PD patients (Schapira, 2007). Toulorge et al. (2016) elegantly compiled a detailed catalog of many mitochondrial bioenergetic deficits in post-mortem PD brain reported in the literature. Other studies have indicated altered mitochondrial biogenesis, mitochondrial fusion-fission homeostasis, defective mitophagy in post-mortem PD brain, PD transgenic animals, or toxin (rotenone, 6-hydroxydopamine, and MPTP) induced models of PD in animals or cultured cells of neural origin (Park et al., 2018; Jiang X. et al., 2019). Peroxisome proliferator-activated receptor-gamma coactivator-1 α (PGC-1α) coordinates the expression levels of many genes needed for mitochondrial biogenesis and electron transport chain activity. The expression of PGC-1α was reported to be decreased in substantia nigra of PD patients, which could be partially attributed to increased cytosine methylation at the promoter region of the PGC-1α gene (Eschbach et al., 2015; Su et al., 2015). PGC-1α regulated nuclear-genes encoding mitochondrial proteins were also downregulated in substantia nigra of the PD brain (Zheng et al., 2010). Familial PD-related genes like *PARKIN, PINK1, DJ1* encode proteins that regulate mitophagy and multiple other mitochondrial functions. The mutations of these genes in experimental genetic models of PD show many mitochondrial anomalies (Gandhi et al., 2009; Irrcher et al., 2010; Heeman et al., 2011; Thomas et al., 2011; Shaltouki et al., 2015; Chung et al., 2016). In a recent study, Zilocchi et al. (2018) reported altered mitochondrial morphology and decreased levels of optic atrophy 1 or OPA1, a mitochondrial fusion protein, and mitochondrial voltage-dependent anion channel (VDAC1 and VDAC2) proteins in substantia nigra of PD brains as well as in dopamine-induced neurodegeneration model in SH-SY5Y cells. Recent investigations also identified various deletion mutations and rearrangements of mtDNA with altered copy numbers in nigral neurons of sporadic PD patients and experimental models of PD, but their significance in PD pathogenesis could not be established as yet (Martín-Jiménez et al., 2020). Altogether an enormous body of data has been generated on mitochondrial anomalies in clinical and experimental PD models, and these have been linked to oxidative stress, neuroinflammation, calcium dysregulation, and finally, neurodegeneration.

#### Oxidative Stress in PD

The accumulation of transition metals like iron and copper, oxidative damage markers of phospholipids, proteins, and DNA, depletion of reduced glutathione (GSH) and increased activity of glutathione degradative enzyme, and elevated levels of peroxiredoxins were reported in substantia nigra of post-mortem PD brains (Jenner, 2003; Ayton and Lei, 2014; Toulorge et al., 2016; Guo et al., 2018). The accumulation of iron in the substantia nigra of PD patients was confirmed both in post-mortem brain samples or by antemortem imaging techniques (Ayton and Lei, 2014; Wang J. Y. et al., 2016). In post-mortem PD brain and toxin-based and genetic models, the key role of reactive oxygen species (ROS) and oxidative stress is apparent in PD pathology (Wu et al., 2003; Varcin et al., 2012; Sanders and Greenamyre, 2013). The accumulation of iron in substantia nigra can lead to ROS generation, especially when the iron is bound to proteins like α-synuclein. Apart from that, the mitochondrial complex I inhibition and other bioenergetic impairment, autoxidation or enzymatic oxidation of dopamine, and activation of microglial NADPH oxidase are also responsible for increased ROS formation in the PD brain (Jana et al., 2011; Bisaglia et al., 2014; Ganguly et al., 2017; Puspita et al., 2017; Guo et al., 2018). In turn, ROS can damage the mitochondrial DNA or electron transport chain components, causing a further increase in ROS production or interact with mitochondrial permeability transition pore to release apoptogenic factors or with the endoplasmic reticulum (ER) to cause Ca^2+^ dysregulation and ER stress (Puspita et al., 2017; Guo et al., 2018). ROS have a complex action on the proteasomal pathway of protein degradation, but with a high level of oxidative stress inactivation of ubiquitinating enzymes, 20S proteasomal catalytic and 19S regulatory units occur (Pajares et al., 2015). Thus increase in ROS may lead to intra-neuronal accumulation of proteins like α-synuclein, which is cleared from the cell in a significant way by the proteasomal pathway (Ebrahimi-Fakhari et al., 2011). In the context of oxidative damage in PD, it is to be mentioned that dopamine oxidation produces ROS as well as highly reactive quinones, which can avidly form conjugates with proteins through thiol residues inactivating their functions, and this process may have important implication in PD pathogenesis (Bisaglia et al., 2014).

#### Inflammation in PD Pathogenesis

The inflammatory response is an essential feature of PD pathogenesis, which has been validated by many published reports over the last several decades. In the striata of post-mortem PD brain, activated microglia, and elevated levels of pro-inflammatory cytokines were demonstrated in multiple studies (Stojkovska et al., 2015; Caggiu et al., 2019). Microglia are primarily responsible for the inflammatory response in the brain. In post-mortem PD brains, the increased number of microglial cells exhibiting activation markers like HLA-DR, ICAM-1, CD 68, and CD23 were reported in multiple studies (Toulorge et al., 2016; Caggiu et al., 2019). The activated microglia liberate a variety of pro-inflammatory cytokines and chemokines. Several studies reported elevated levels of TNF-α, IL1β, IL6, IFN-γ, TGF-β, and others in the striatum, substantia nigra, and CSF from post-mortem PD brains (Mogi et al., 1996; Nagatsu et al., 2000; Toulorge et al., 2016; Caggiu et al., 2019; Pajares et al., 2020). In conformity with these post-mortem findings, an upregulation of pro-inflammatory cytokines was observed in the brain of 6-hydroxydopamine or MPTP-based models of PD (Pajares et al., 2020). Microglial ROS production through activation of NADPH oxidase is characteristic of the inflammatory response, which is also enhanced in the substantia nigra of post-mortem PD brain as well as in the brain of MPTP induced mice model of PD (Belarbi et al., 2017). The activation of microglia in the brain occurs through many triggers like aggregated α-synuclein, pesticides, MPTP, bacterial lipopolysaccharide (LPS), and cytokines, which have implications in dopaminergic neuronal death in PD as observed in different experimental systems (Sanchez-Guajardo et al., 2015; Caggiu et al., 2019; Lee et al., 2019; Zhao et al., 2019). It is already established that elevated peripheral cytokines can lead to the activation of brain microglia through neural and humoral pathways (Dilger and Johnson, 2008). Thus, it is interesting that a meta-analysis confirmed elevated levels of circulating pro-inflammatory cytokines in sporadic PD, and this may link systemic inflammatory and immune response to PD pathogenesis (Qin et al., 2016). Apart from the central role of microglia in brain inflammatory response, astrocytes have been implicated recently in the latter process, but their importance in PD pathogenesis is not clearly established (Toulorge et al., 2016; Pajares et al., 2020). On the other hand, activated microglia can affect the blood-brain barrier allowing the invasion of the brain by peripheral monocytes and lymphocytes, which might also contribute to the inflammatory response in the brain (Pajares et al., 2020).

#### α-Synucleinopathy and PD Pathogenesis

The importance of α-synuclein in the pathogenesis of PD is indicated by the fact that it is the most abundant protein component of LBs and LNs. Moreover, point mutations (A53T, A30P, E46K, G51D, and several others) and multiplications (duplications or triplications) of the *SNCA* gene (coding for α-synuclein) have been reported in individuals with familial PD with autosomal dominance (Polymeropoulos et al., 1997; Krüger et al., 1998; Zarranz et al., 2004; Klein and Westenberger, 2012; Kiely et al., 2013). This small acidic protein of 140 amino acids is expressed abundantly in the brain and located at the presynaptic terminals where it is presumably involved in the vesicular transport of neurotransmitters (Stefanis, 2012; Lashuel et al., 2013). This intrinsically unfolded protein takes up partially helical structures in contact with biomembranes (Stefanis, 2012). Under certain conditions, α-synuclein attains β-conformation and undergoes aggregation to form soluble oligomers of various types and finally insoluble fibrils (Breydo et al., 2012; Stefanis, 2012). α-Synuclein undergoes different post-translational modifications such as phosphorylation, nitration, acetylation, *o*-GlcNAcylation (*N*-acetylglucosamine attached through serine hydroxyl groups), truncation, and ubiquitination, and some of these may modify the aggregation, membrane-binding, metal-binding, and other properties of this protein (Oueslati, 2016; Zhang et al., 2019; He et al., 2021). Though α-synuclein can undergo both serine and tyrosine phosphorylation at multiple sites through different kinases, the phosphorylated α-synuclein at serine 129 has been linked to toxicity in different studies (Oueslati, 2016; Arawaka et al., 2017; Zhang et al., 2019).

Transgenic organisms (flies, worms, and mice) overexpressing human wild-type or mutant human α-synuclein exhibit dopaminergic neurodegeneration, LB pathology, and motor deficits to varying extents (Breydo et al., 2012; Stefanis, 2012). Accumulated evidence from a large number of experimental studies clearly demonstrates that the administration of human α-synuclein protein or lentivirus or recombinant adeno-associated virus-carrying wild or mutant human α-synuclein gene in the substantia nigra of rodents and monkeys can lead to progressive neurodegeneration and motor deficits, and these studies have been well-summarized in several reviews (Dehay and Fernagut, 2016; Ganguly et al., 2018). Likewise, catecholaminergic cell lines manipulated genetically or pharmacologically in culture to express high levels of wild or mutant α-synuclein undergo degeneration under basal conditions or cellular stress (Zhu et al., 2012; Ramalingam et al., 2019; Ganguly et al., 2020). The varied mechanisms of α-synuclein toxicity altering mitochondrial functions and endoplasmic reticulum (ER)-Golgi transport or causing ER -stress and autophagic impairment in different model systems are available in several reviews (Stefanis, 2012; Lashuel et al., 2013; Ganguly et al., 2018; Fields et al., 2019). Some of the recent studies have provided further insights into α-synuclein toxicity in different model systems, which could be important in the context of PD pathogenesis. For example, mitochondria and ER remain tethered to each other at certain zones through interactions of a mitochondrial outer membrane protein and an integral protein of ER. Paillusson et al. (2017) showed that over-expression of α-synuclein (wild or mutated) disrupted the interactions of mitochondria and ER, leading to defective Ca^2+^ exchange between these two compartments and decreased mitochondrial ATP production. Similarly, Ganguly et al. (2020) suggested that accumulated α-synuclein in SH-SY5Y cells exposed to iron interacted with mitochondrial permeability transition pore components to cause mitochondrial depolarization and loss of ATP production. This study also showed how iron-dependent oxidative inactivation of Parkin, an E3 ubiquitin ligase, could lead to intracellular accumulation of α-synuclein presumably by preventing its degradation in the proteasomal pathway. Consistent with this idea, Chung et al. (2020) showed that a cell-permeable form of Parkin could prevent the accumulation of α-synuclein aggregates and fibrils when SH-SY5Y cells overexpressing α-synuclein were exposed to a mitochondrial toxin like rotenone. This study also demonstrated that cell-permeable Parkin could remove damaged mitochondria by mitophagy and promote mitochondrial biogenesis. The authors further confirmed the findings in toxin-based animal models of PD (Chung et al., 2020). In another study using Drosophila and mice models of α-synucleinopathy, it was demonstrated that α-synuclein interacted with cytoskeletal proteins spectrin and actin to cause mitochondrial morphological and functional alterations through mislocalization of a mitochondrial fission protein (Ordonez et al., 2018). Ludtmann et al. (2018) using inducible pluripotent stem cells (iPSC)-derived neurons with triplication of *SNCA* gene showed that oligomeric α-synuclein through inhibition of mitochondrial complex I activity caused oxidation of a subunit of ATP synthase leading to the opening of mitochondrial permeability transition pore with consequent mitochondrial swelling and cell death. In a similar model of iPSC-derived cortical neurons carrying triplicate *SNCA* gene, increased expressions (mRNA and protein) of ER stress and unfolded protein response (UPR) markers could be observed (Heman-Ackah et al., 2017). In a Drosophila model of α-synucleinopathy and neurodegeneration, the expression of human α-synuclein caused impairment of autophagolysosomal system leading to an accumulation of autophagosomes and mitophagosomes, and the process involved abnormal stabilization of actin filaments (Sarkar et al., 2021). Likewise, in PC12 cells, the overexpression of human wild-type or mutant α-synuclein prevented starvation-induced autophagic vesicle formation with downregulation of autophagic markers (Wang K. et al., 2016). This plethora of studies identifying different potential mechanisms of α-synuclein toxicity have significant implications in PD pathogenesis, but so far, no consensus mechanism of α-synuclein mediated neurodegeneration has emerged.

## Biomarkers of PD

Multiple studies have reported the usefulness of several biochemical or imaging biomarkers in the differential diagnosis of PD. However, no single biomarker is specific enough for routine use in the diagnostic or prognostic evaluation in clinical cases of PD. On the other hand, the genetic biomarkers are mutations in defined genes or susceptibility loci that have been useful only in establishing the familial nature of PD.

### Imaging Biomarkers

Several imaging techniques such as magnetic resonance imaging (MRI), transcranial sonography (TCS), single-photon emission computed tomography (SPECT), and positron emission tomography (PET) have been used to identify structural changes in the substantia nigra. These techniques can estimate the levels of dopamine transporter (DAT), vesicular monoamine transporter (VMAT), post-synaptic dopamine receptors, aromatic amino acid decarboxylase activity, abnormal accumulation of proteins (α-synuclein, and tau) or iron in the mid-brain or other areas. Such measurements have assisted in understanding the pathogenesis of the disease or its progression or effects of drugs on it (Niethammer et al., 2012; Wang L. et al., 2012). However, the results from such studies are not unequivocal to recommend any particular imaging biomarker for PD diagnosis. High-resolution MRI can identify a unique cluster of dopaminergic neurons in substantia nigra, called nigrosome-1, and the ‘swallow-tail’ appearance of healthy nigrosome-1 is lost in PD (Schwarz et al., 2014). It appears that though routine structural MRI may not be of much use in the diagnosis of PD, advanced MRI techniques such as diffusion imaging and susceptibility-weighted imaging for iron-load could be useful for supportive diagnosis of PD (Pyatigorskaya et al., 2014). The efficacy of TCS in the diagnosis of PD has also been examined, but in 196 consecutive cases of clinically unclear parkinsonism, the diagnostic accuracy of TCS of substantia nigra in identifying early cases of PD could not be established (Bouwmans et al., 2013). The imaging of DAT with PET or SPECT scan has again been suggested as only a piece of supportive evidence in favor of PD diagnosis (Brooks, 2016). Nevertheless, ^18^F-labeled L-6-fluoro-3,4-dihydroxyphenylalanine (^18^F-DOPA) PET-scan could eventually become an important diagnostic tool for the differential diagnosis of PD (Calabria et al., 2016). Further, an ^18^F-labeled 2-deoxy-2-fluoro-D-glucose (^18^F-FDG) PET scan is being accepted as a good method for differential diagnosis of PD from other parkinsonian disorders or risk assessment of cognitive impairment in PD (Meyer et al., 2017).

### Genetic Biomarkers

The familial nature of PD was first identified in some Italian and Greek families where an autosomal dominant inheritance of the disease was attributed to mutations in the *SNCA* gene (coding for α-synuclein) present in the long arm of chromosome 4 (Nussbaum and Polymeropoulos, 1997; Polymeropoulos et al., 1997). From that time onward, based on linkage analysis, exome sequencing, and genome-wide association studies (GWAS), nearly 28 chromosomal loci have been implicated in genetic forms of PD, and 18 of them have been included in the PARK family and named as PARK 1/4, PARK 2, PARK 3, PARK 5, PARK 6 and so on (Klein and Westenberger, 2012). Within these loci several genes have been identified in which mutations can lead to monogenic forms of familial PD with either autosomal dominant or recessive inheritance or some complex disorders with parkinsonism as an associated component; in such cases, the mutations by themselves are sufficient for causing the disease (Lesage and Brice, 2009; Klein and Westenberger, 2012). The other chromosomal loci without identified genes may also cause familial PD, while some other loci probably represent susceptibility or genetic risk for developing PD (Pankratz and Foroud, 2007; Lesage and Brice, 2009; Klein and Westenberger, 2012). Among the monogenic forms of PD, mutations in *LRRK2* (PARK8) coding for leucine-rich repeat kinase 2 protein and *SNCA* (PARK1/4) coding for α-synuclein cause autosomal dominant PD; LRRK2 mutations are responsible for a significant percentage of familial PD, but SNCA mutations are rare (Pankratz and Foroud, 2007; Lesage and Brice, 2009; Klein and Westenberger, 2012). However, duplication or triplication of SNCA locus is also reported to cause familial PD with an earlier onset of the disease seen in those with triplication (Singleton et al., 2003; Book et al., 2018). Mutations in *PARKIN* (PARK2) coding for an E3 ubiquitin ligase enzyme called Parkin, and *PINK1* (PARK6) coding for a protein kinase called phosphatase and tensin homolog (PTEN)-induced protein kinase 1 cause autosomal recessive disease accounting for the majority of familial PD cases, while mutations in *DJ-1* (PARK7) are seen in rare forms of familial PD with autosomal recessive inheritance (Pankratz and Foroud, 2007; Klein and Westenberger, 2012). As in PARK 1/4, PARK2, PARK7, some of the gene mutations cause early-onset PD (sometimes even juvenile-onset) with aggressive progress, while typical late-onset PD with slow progress as in sporadic disease is caused by other mutations in PARK8 (LRRK2), PARK13, and PARK17. Mutations in PARK9 (*ATP13A2*) cause a complex phenotype with associated parkinsonism (Klein and Westenberger, 2012). The inheritance patterns of monogenic familial PD get complicated because many mutations have incomplete or variable penetrance and expressivity, making it difficult to identify some family members with the disease. In addition, a phenocopy may appear in the family, which means a person with similar clinical features of the disease caused by some unknown environmental factors or by a different genetic mechanism (Klein and Westenberger, 2012).

It is also important to analyze how genes related to monogenic familial PD can also be important in sporadic PD. Besides specific mutations causing familial PD, in a given population, there are other genetic variations (polymorphisms) within such genes, their promoters, or in other sites in the gene loci, which may pose as susceptibility or genetic risk factors in the genesis of sporadic PD. The heterozygous carriers of autosomal recessive PD could also be at a higher risk of developing sporadic PD (Lesage and Brice, 2009; Klein and Westenberger, 2012; Mullin and Schapira, 2015). Such genetic variants or heterozygous mutations of SNCA, LRRK2, PARKIN, PINK1, and GBP (coding for lysosomal β-glucocerebrosidase) genes have been implicated in sporadic PD, emphasizing the importance of gene-environment interactions in this disease (West et al., 2002; Abou-Sleiman et al., 2006; Winkler et al., 2007; Lesage and Brice, 2009; Ross et al., 2011; Mullin and Schapira, 2015; Do et al., 2019). Further, the familial PD-related genes and their protein products have helped us understand the molecular mechanisms underlying PD pathogenesis, and much experimental work is available to explain PD pathogenesis through the involvement of α-synuclein, Parkin, PINK1, and DJ-1. However, the identification of mutations related to monogenic PD is beneficial clinically only in a small set of patients with positive family history and early onset of the disease or presenting with some complex phenotype in addition to parkinsonism. The incomplete penetrance and expressivity of these mutations and the fact that some PARK loci are just indicative of PD susceptibility, genetic counseling, or routine genetic testing of all persons having a positive family history of PD is not recommended. As far as the vast majority of sporadic PD cases are concerned, the identification of susceptibility variants or heterozygous mutations of familial PD genes holds some promise for the future development of a genetic biomarker for PD, but for this, many large-scale statistically powered GWAS or other kinds of analyses would be necessary.

### Biochemical Biomarkers

The biochemical biomarkers for PD include a broad range of molecules that have been analyzed in CSF, blood, or other biological fluids. These include dopamine and its catabolites, several other types of amine or amino acid neurotransmitters, neuropeptides, oxidative damage markers, purine catabolite like uric acid, inflammatory markers, neurotrophic factors like brain-derived neurotrophic factor (BDNF), microRNAs, and several proteins known to be associated with the pathogenesis of PD and other neurodegenerative disorders, and all these have been discussed elaborately in multiple reviews and meta-analyses (Jiménez-Jiménez et al., 2014; Emamzadeh and Surguchov, 2018; He et al., 2018; Wei et al., 2018; Jiang L. et al., 2019; Katayama et al., 2020). To avoid repetition and because no clinically useful blood or CSF biochemical biomarker specific for PD is still available, we will only briefly discuss a few of these biomarkers. Oxidative stress and inflammatory markers, are not specific for PD and are usually altered in multiple other neurological diseases where oxidative damage and inflammation are involved in the disease pathogenesis. BDNF, a member of the neurotrophin family, is an important regulator of neuronal differentiation, proliferation, and survival, and in several experimental models of PD, the neuroprotective role of BDNF was earlier demonstrated (Palasz et al., 2020). However, the alterations in BDNF levels in CSF and blood in PD patients as reported in multiple studies are complex and inconsistent, and its biomarker potential in this disorder is not established (Nagatsu et al., 2000; Salehi and Mashayekhi, 2009; Ventriglia et al., 2013; Jiang L. et al., 2019). The purine derivative and the potent antioxidant uric acid (measured both in the serum and CSF) have been explored as a candidate risk factor and a diagnostic and prognostic biomarker for PD (Cipriani et al., 2010; Paganoni and Schwarzschild, 2017). A meta-analysis of eligible studies on serum uric acid level as a diagnostic biomarker has shown that PD is associated with a low uric acid level in serum (Wen et al., 2017). Dopamine and its catabolites like dihydroxy phenylacetic acid (DOPAC) and homovanillic acid (HVA) in CSF have been analyzed for a long time to assess the loss of central dopaminergic functions and to correlate it with the motor impairment in PD. The CSF levels of HVA and DOPAC were reported to be elevated in PD patients at early stages, and the values increased further with the degree of motor impairment (Stefani et al., 2017). In contrast, a recent follow-up study showed that subjects with several PD-related risk factors had a higher chance of being afflicted with the disease if their baseline CSF levels of dopamine and DOPAC were low (Goldstein et al., 2018). In another study, the CSF level of HVA could not differentiate PD patients from the controls, but the ratio of xanthine/HVA clearly separated the two groups (LeWitt et al., 2011). However, many earlier studies reported a decline in CSF HVA levels in PD, and these studies as well as the changes in serotonin catabolites in PD have been well-reviewed elsewhere (Jiménez-Jiménez et al., 2014).

Metabolomics has opened up new avenues of research in biomarker identification for pathological conditions. In this approach, instead of a hypothesis-driven targeted search for a biochemical biomarker for disease, complete profiles of the metabolites are compared between the control and diseased subjects employing primarily a liquid chromatography-mass spectrometry (LC-MS) based separation-detection system. Multiple studies have adopted this approach using blood or CSF to identify sets of molecules to distinguish PD from control subjects. These metabolites are sugars, fatty acids, amino acids, peptides or molecules related to phosphoglyceride and sphingolipid metabolism, amino acid metabolism, mitochondrial functions, energy metabolism, and glutathione metabolism (Trupp et al., 2014; Wuolikainen et al., 2016; Stoessel et al., 2018; Willkommen et al., 2018; Zhao et al., 2018). Although a promising approach, a metabolomics-based search for a set of definitive and clinically useful biomarkers for the diagnosis of PD is yet to be achieved.

Identifying protein biomarkers of PD from familial PD gene products, which could help in the diagnosis or prognostic evaluation of the disease, forms an important area of PD research, and in this context, we will focus our attention on α-synuclein. Table 1 summarizes the different types of PD biomarkers with their advantages and limitations.

**TABLE 1:** Biomarkers in Parkinson’s disease (PD).

Biomarker	Techniques used	Targets analysed	Advantages	Limitations
Imaging biomarkers	Magnetic resonance imaging (MRI), transcranial sonography (TCS), single-photon emission computed tomography (SPECT), positron emission tomography (PET)	Dopamine transporter (DAT), vesicular monoamine transporter (VMAT), post-synaptic dopamine receptors, aromatic amino acid decarboxylase activity, accumulation of proteins (α-synuclein, tau), iron, etc.	Non-invasive. Good supportive evidence for diagnosis. ^18^F-DOPA PET scan and ^18^F-FDG PET scan showing great promise as diagnostic tools.	No unique biomarker found yet. Expensive and may not be easily available.
Genetic biomarkers	Linkage analysis Exome sequencing Genome-wide association studies (GWAS)	Mutations in genes and susceptibility loci in chromosomes (PARK family)	Confirmation of genetic nature of PD in patients with positive family history or with an early onset of the disease or having a complex phenotype in addition to PD.	Useful only for a small subset of PD patients. Due to incomplete penetrance and expressivity of the mutations, the genetic testing/counseling not recommended routinely.
Biochemical markers	HPLC, immuno-assays, biochemical assays, immuno-blotting, immuno-histochemistry, LC-MS	Dopamine metabolites (DOPAC, HVA), oxidative damage markers, inflammatory markers, miscellaneous markers (amino acid derivatives, uric acid, BDNF, peptides, miRNAs) Metabolomic profile, Protein markers (α-synuclein, LRRK2, DJ-1, tau, etc.)	Some markers can be assessed in easily accessible biofluids or tissues. Automated assays for a large number of samples possible in many cases. Some markers are related to disease pathogenesis directly. α-Synuclein is a promising protein biomarker. Metabolomic profiling could become an emerging technique.	Some biomarkers like oxidative damage or inflammatory markers are non-specific. Others like uric acid not directly related to pathology. No single unique biomarker is still available.

*BDNF, brain-derived neurotrophic factor; DA, dopamine; DOPAC, dihydroxy phenyl acetic acid; HPLC, high performance liquid chromatography; HVA, homovanillic acid; LC-MS, liquid chromatography-mass spectrometry; LRRK2, leucine rich repeat kinase-2; ^18^F-DOPA, ^18^F-labeled L-6-fluoro-3,4-dihydroxyphenylalanine; ^18^F-FDG, ^18^F-labeled 2-deoxy-2-fluoro-D-glucose.*

## α-Synuclein as a Biomarker

Because of the involvement of α-synuclein in PD pathogenesis and its presence in the CSF, blood, and other body fluids, the biomarker potential of this protein has been examined extensively in PD as well as other synucleinopathies. However, α-synuclein is expressed by many different tissues, and there is a bi-directional movement of this protein between blood and the brain (Sui et al., 2014). Thus, it may be difficult to interpret if the alterations in the levels of α-synuclein in body fluids reflect PD pathology in the brain. Additionally, the protein can exist in monomeric and multiple oligomeric forms as well as post-translationally modified forms which can add to the complexity of measurement of this protein.

### α-Synuclein in Cerebrospinal Fluid (CSF)

The CSF is an ideal body fluid to look for PD biomarkers as it is expected to provide the metabolic-pathological profile of the CNS. Multiple studies have investigated the alterations in the levels of α-synuclein in the CSF, but the results are varied. While many studies have reported a lower mean total α-synuclein in CSF of PD patients with respect to age-matched controls or other neurological controls, a few studies failed to find any significant difference between the PD and control groups (Tokuda et al., 2006; Mollenhauer et al., 2008, 2011, 2013; Ohrfelt et al., 2009; Hong et al., 2010; Park et al., 2011; Parnetti et al., 2011; Shi et al., 2011; Hall et al., 2012; Toledo et al., 2013; van Dijk et al., 2013; Hansson et al., 2014; Wennström et al., 2013). Most studies, systematic reviews, and meta-analyses which examined the validity of α-synuclein in the CSF as a putative biomarker for PD emphasized that a decreased level of α-synuclein in CSF reliably separated PD from control subjects, but the specificity of this measurement was low (Mollenhauer et al., 2011, 2013; Aerts et al., 2012; Gao et al., 2015; Zhou et al., 2015; Chahine et al., 2020). Further, in the differential diagnosis of PD from other movement disorders like MSA, progressive supranuclear palsy, corticobasal degeneration, and vascular parkinsonism, the utility of α-synuclein in CSF as a single biomarker is not established (Aerts et al., 2012; Gao et al., 2015; Zhou et al., 2015). Other workers attempted to measure oligomeric α-synuclein in CSF to examine its suitability as a biomarker for PD. Thus, increased levels of α-synuclein oligomers (o-α-syn) in CSF have been reported in PD with dementia compared to age-matched controls. Similarly, others have reported decreased levels of α-synuclein, raised levels of o-α-syn and increased o-α-syn/total α-synuclein ratio in the CSF of PD patients compared to that in age-matched control subjects or various non-PD neurological controls (Tokuda et al., 2010; Hansson et al., 2014; Park et al., 2011; Parnetti et al., 2014a,b; van Steenoven et al., 2018). Combined measurements of the ratios of o-α-synuclein/total α-synuclein and β-glucocerebrosidase activity or Aβ42/total-tau improved the specificity of PD diagnosis (Parnetti et al., 2014a,b). Likewise, the ratio of tau to α-synuclein in CSF has been claimed to be of high specificity in distinguishing PD from other parkinsonian syndromes or neurological controls (Parnetti et al., 2011; Heegaard et al., 2014). The measurement of CSF α-synuclein in combination with FMS-like tyrosine kinase 3 ligand (FLT3L) or the percentage phosphorylated tau (p-tau) clearly distinguished PD from MSA in a study (Shi et al., 2011). A recent study employed single-molecule array (Simoa), an ultra-sensitive technology, to quantitate very low concentrations (picomolar to femtomolar) of α-synuclein protofibrils (PFs) in CSF of PD patients and observed an increased concentration of α-synuclein PFs in PD patients (von Euler Chelpin et al., 2020). The increased baseline level of misfolded α-synuclein aggregate in CSF (measured by protein misfolding cyclic amplification technique) has been reported in a follow-up study as a risk factor for the development of dementia in PD (Ning et al., 2019). These varied α-synuclein levels and forms in PD patients reported by various study groups might be the result of changes in the secretion, solubility, and aggregation of the protein, thereby affecting its overall turnover.

Post-translational modifications of α-synuclein affect its solubility and make it prone to aggregation. The most common pathological post-translational modification of α-synuclein is phosphorylation, particularly at Ser129. Phosphorylated α-synuclein (pS129) has been identified in CSF by mass spectrometry. A cross-sectional study reported higher pS129 in PD patients compared to healthy controls or MSA or PSP; the ratio of pS129/total α-synuclein is significantly higher in PD than in healthy controls or PSP (Wang Y. et al., 2012). Unlike the CSF levels of total α-synuclein, the levels of pS129 have a weak correlation with PD severity (Wang Y. et al., 2012). A study by the same group in the longitudinal DATATOP cohort (deprenyl and tocopherol antioxidative therapy of Parkinsonism) which included individuals with early PD in a placebo-controlled clinical trial reported increased CSF pS129 and pS129/total α-synuclein ratio over 2 years of disease progression (Stewart et al., 2015). Another important study corroborated the earlier findings of decreased α-synuclein and elevated levels of o-α-syn and pS129 in CSF in PD than that in controls, and further demonstrated that the combined measurements of o-α-syn/total α-synuclein and pS129/total α-synuclein could clearly improve the discrimination of PD from controls (Majbour et al., 2016). However, this study did not find any correlation of the CSF level of pS129 with the severity of PD and instead showed a modest inverse correlation with o-α-syn levels with the latter.

Although CSF appears to be an ideal body fluid to look for PD biomarkers, as it is in direct contact with the brain and the spinal cord, it may be mentioned that the process of obtaining CSF involves an invasive, painful procedure requiring skilled staff, and there may be additional technical and ethical issues related to CSF collection for a particular disease.

### α-Synuclein as a Blood Biomarker of PD

The easy accessibility of blood makes it an important body fluid to monitor biomarker levels of disease for diagnostic or prognostic evaluation. α-Synuclein levels have been examined in plasma, serum, erythrocytes, and even in peripheral blood mononuclear cells in PD. The level of α-synuclein in plasma exceeds many times that of CSF, and the major source of plasma or serum α-synuclein is the erythrocytes (Barbour et al., 2008). In familial PD patients with SNCA multiplications, high levels of α-synuclein in blood were reported (Miller et al., 2004). One recent case-control study reported increased levels of α-synuclein in the peripheral circulation (plasma and serum) in PD with respect to age-matched healthy controls, and further, the serum levels correlated with the clinical severity of the disease (Chang et al., 2020). Other studies also observed increased levels of α-synuclein in the plasma of PD patients than that in controls, but the correlation of this parameter with the degree of motor disability was variably reported (Lee et al., 2006; Ding et al., 2017; Lin et al., 2017; Wang et al., 2019). A small study with 34 PD patients and 27 controls earlier reported an increased plasma level of soluble oligomeric α-synuclein in the patients’ group (El-Agnaf et al., 2006). However, several other studies either showed decreased levels of α-synuclein in plasma in PD subjects than that in controls or failed to observe any significant difference in this parameter between the two groups (Li et al., 2007; Shi et al., 2010). In a longitudinal study, PD patients were followed for up to 20 years after initial presentations and plasma total α-synuclein and phosphorylated α-synuclein or pS129 were measured repeatedly (Foulds et al., 2013). Plasma levels of pS129 but not total α-synuclein were higher in PD subjects compared to healthy controls on the first presentation, but during the follow-up period of the PD patients, the levels of total α-synuclein in the plasma increased and that of pS129 remained unaltered (Foulds et al., 2013). These somewhat inconsistent results could be due to technical reasons such as contamination from erythrocyte α-synuclein as a result of hemolysis, different assay techniques employed, and the differential ability of the antibodies to bind to different forms of α-synuclein. In contrast to such studies, attempts have been made to validate the biomarker potential of erythrocyte α-synuclein in PD. In a recent extensive study, total and aggregated α-synuclein and also pS129 were separately measured in the membrane, and cytosolic fractions of erythrocytes; significantly increased levels of total and aggregated α-synuclein in the membrane fraction and remarkably high levels of pS129 in cytosolic fraction were observed in PD cases in comparison to that in healthy controls (Tian et al., 2019). Likewise, another large study of 334 healthy controls, 333 PD and 114 MSA patients, erythrocyte α-synuclein oligomers or o-α-syn were found to be much elevated in PD patients compared to that in other two groups, and apparently, this measurement could differentiate PD patients from control or MSA subjects with good sensitivity and specificity (Li et al., 2019). This study further showed in rat models that an increase in erythrocyte o-α-syn reflected an increased brain level of α-synuclein. An increased level of erythrocyte α-synuclein with respect to healthy controls was also reported in PD patients in another study which, however, showed that higher plasma levels of α-synuclein in PD than controls had better positive predictive value and stronger correlation with disease severity than erythrocyte α-synuclein levels (Wang et al., 2019). The erythrocyte levels of α-synuclein dimers were higher in idiopathic PD and those with GBA mutations than in age and sex-matched controls (Papagiannakis et al., 2018). Likewise, a decreased ratio of total to proteinase K resistant α-synuclein (phospholipid-bound α-synuclein) in red blood cells was shown to discriminate PD patients from healthy controls (Abd-Elhadi et al., 2015). The same group in a later study measured multiple forms of α-synuclein consisting of total α-synuclein, proteinase-K resistant α-synuclein, oxidized α-synuclein, and pS129 and demonstrated that the higher levels of total and proteinase K resistant α-synuclein and pS129 differentiated PD patients with motor symptoms (without dementia) from healthy controls with a high degree of accuracy (Abd-Elhadi et al., 2019). On the other hand, a recent study estimated the levels of total α-synuclein, o-α-syn, total-tau, phosphorylated tau, α-synuclein-amyloid β42 complex, and α-synuclein-tau complex in red blood cells of PD patients; decreased total α-synuclein and increased levels of o-α-syn, phosphorylated-tau, and α-synuclein-amyloid β42 complex were observed in PD subjects with respect to controls (Daniele et al., 2018). This study also suggested that the level of α-synuclein-amyloid β42 complex in red blood cells correlated well with the degree of disease severity. Another extensive study detected different post-translationally modified α-synuclein in the lysate of red blood cells such as different types of phosphorylated α-synuclein apart from pS129, nitrated or glycated or small ubiquitin-like modifier or SUMO-ylated α-synuclein, and showed that phosphorylated, nitrated, and glycated α-synucleins were elevated and SUMO-ylated α-synuclein was decreased in PD patients with respect to that in controls (Miranda et al., 2017).

α-Synuclein in oligomerized or aggregated form is known to be released outside from the neurons packed in extracellular vesicles (EVs), and such EVs may be responsible for the neuron-to-neuron transfer of α-synuclein and the spread of PD pathogenesis in the brain (Lööv et al., 2016; Yu et al., 2020). The EVs from the brain likewise may carry toxic oligomeric α-synuclein to the peripheral circulation, and some studies examined the biomarker potential of EV-α-synuclein in blood (Vandendriessche et al., 2020; Yu et al., 2020). For the latter purpose, α-synuclein present in neuron-derived EVs, positive for neuronal cell adhesion molecule LCAM1, were analyzed (Lööv et al., 2016; Vandendriessche et al., 2020). Thus, a large study measured α-synuclein content in plasma as well as in LCAM1 positive EVs isolated from the blood of 267 PD and 215 controls; the plasma levels of α-synuclein did not differ in the groups, but LCAM1 positive EV-α-synuclein was significantly higher in the PD cases compared to that in the controls (Shi et al., 2014). Similarly, increased α-synuclein content of neuron-derived EVs in serum of PD patients could differentiate them from the controls not only in the presence of motor deficits but even during the prodromal phase (Jiang et al., 2020). The authors further showed that simultaneous measurements of α-synuclein and clusterin in neuron-derived EVs could distinguish PD from several other parkinsonian disorders.

### α-Synuclein in Saliva

α-Synuclein has been detected in the saliva of PD patients. A decrease in the level of total α-synuclein in the saliva of PD patients in comparison to controls has been reported from several studies (Devic et al., 2011; Al-Nimer et al., 2014). Vivacqua et al. (2016) reported a significant increase in o-α-syn and o-α-syn/total α-synuclein ratio, but a decreased level of total α-synuclein in the saliva of PD patients. The authors suggested that the decrease in total α-synuclein in saliva in PD subjects could partly be accounted for by the oligomerization of monomeric α-synuclein. Similar findings were reported by Shaheen et al. (2020), which also showed a positive correlation between the level of o-α-syn and disease duration; further, in PD patients, higher levels of o-α-syn were seen in bradykinesia and rigidity-dominant than in tremor-dominant phenotypes. Another study measured the salivary total α-synuclein and o-α-syn between controls and PD cases and analyzed single nucleotide polymorphisms or SNP variants of *SNCA* in the PD group (Kang et al., 2016). The study failed to observe any significant difference in salivary total α-synuclein level between the PD and healthy control groups, but the o-α-syn level was significantly higher in the PD group. This study further showed that salivary level of total α-synuclein decreased with age with no correlation with disease duration or severity but depended upon the SNP variant of *SNCA* (Kang et al., 2016). A meta-analysis of existing studies on salivary α-synuclein as a biomarker for PD concluded that the association of increased o-α-syn in saliva with PD looks significant, but no such association with PD could be established for salivary total α-synuclein (Bougea et al., 2019). In various other tissues like the enteric nervous system, retina, and skin, α-synucleinopathy is being investigated vigorously to understand various non-motors features of PD and overall pathology of the disease, but the biomarker implications of these studies are not clear at this moment

### α-Synuclein in Skin

Wang et al. (2013) compared the deposition of α-synuclein in the cutaneous nerves between PD patients and healthy controls. The study reported increased deposits of α-synuclein, detected by immunohistochemistry followed by confocal imaging, in the cutaneous sympathetic nerves (both cholinergic and adrenergic) in PD subjects with higher deposition observed in the advanced cases of the disease and with more autonomic dysfunction. In a similar study, Gibbons et al. (2016) reported a higher deposit of α-synuclein in the skin biopsy samples of PD patients than control subjects with a greater deposition in patients of more severity or longer duration or with autonomic dysfunction. The authors further observed that α-synuclein deposition was more in sympathetic adrenergic fibers than in cholinergic ones. In a recent study, the oligomeric form of α-synuclein or o-α-syn was detected for the first time in autonomic nerve terminals in skin biopsy samples using a proximity ligation assay; a significantly higher deposition of o-α-syn was seen in 38 consecutive PD patients in comparison to 29 consecutive healthy controls (Mazzetti et al., 2020). Interestingly, the authors of this study further analyzed o-α-syn deposition in skin biopsy samples of 19 pairs of monozygotic twins discordant for PD. Within this cohort of twins, a higher deposition of o-α-syn was noticed in PD patients.

Several studies demonstrated the deposition of pS129 in the cutaneous nerves in PD subjects in comparison to that in control subjects (Donadio et al., 2014, 2016). Zange et al. (2015) demonstrated that deposition of pS129 in skin sympathetic nerve fibers could distinguish PD from patients of MSA and essential tremor. Doppler et al. (2014) in their study observed the deposition of phosphorylated α-synuclein in skin nerve fibers in PD patients predominantly in autonomic nerves supplying blood vessels, erector pili muscles and sweat glands, but some deposition also occurred in somatosensory and other types of nerve fibers in the epidermis and dermis. This study suggested that the accumulation of phosphorylated α-synuclein caused peripheral neurodegeneration in PD. Another group utilized two new techniques, real-time quaking-induced conversion and protein misfolding cyclic amplification assays, to measure the aggregation-seeding activity of phosphorylatexd α-synuclein or pS129 in tissue samples (Wang et al., 2021). The authors then analyzed the aggregation-seeding activity of pS129 in autopsied skin samples from PD and non-PD control cadavers as well as skin biopsy samples from living PD patients and non-PD control subjects (Wang et al., 2021). A higher aggregation-seeding activity of pS129 was seen in skin samples from PD than that in non-PD controls in this study.

### α-Synuclein in the Enteric Nervous System (ENS)

Most PD patients exhibit non-motor symptoms, including autonomic dysfunction of the gastrointestinal tract. The detection of LBs in the ENS of PD patients was made very early (Qualman et al., 1984). They identified these inclusions in the esophagus and colon of two PD patients with dysphagia (Qualman et al., 1984). Wakabayashi et al. (1988, 1990) reported in several publications the presence of LBs in Auerbach’s and Meissner’s plexuses and paravertebral and coeliac sympathetic ganglia in the human ENS. Other studies further confirmed the accumulation and aggregation of α-synuclein in the gut mucosa of PD patients (Braak et al., 2006; Lebouvier et al., 2010; Gold et al., 2013; Sánchez-Ferro et al., 2014). The aggregates of α-synuclein in the gastro-intestinal mucosa also contained a significant amount of the phosphorylated protein (Lebouvier et al., 2010; Barrenschee et al., 2017; Beck et al., 2020). A large-scale study demonstrated the accumulation of α-synuclein in mucosal biopsy samples from the gastrointestinal tract in the pre-motor phase of PD (Hilton et al., 2013). The aggregation of α-synuclein in ENS could be responsible for the enteric abnormality observed in clinical PD cases. It also strengthens the hypothesis that PD may begin peripherally in the gut wall and then proceed toward the central nervous system. As a diagnostic biomarker of PD, the identification of α-synuclein pathology in mucosal biopsy samples from the gastrointestinal tract could be important as well. α-Synuclein aggregates are frequent and of a higher degree in PD patients as compared to age-matched healthy controls (Lebouvier et al., 2010; Forsyth et al., 2011; Shannon et al., 2012; Gold et al., 2013; Sánchez-Ferro et al., 2014; Yan et al., 2018). However, other studies with enteric tissue biopsy samples identified α-synuclein immunoreactivity in normal aged persons or could not find any difference in α-synuclein accumulation between PD patients and controls or observed increased immunoreactivity of phosphorylated but not total α-synuclein in PD subjects (Böttner et al., 2012; Chung et al., 2015; Lee et al., 2018; Beck et al., 2020). Another retrospective study with post-mortem gastro-intestinal mucosal samples of PD patients and controls even claimed decreased deposition of α-synuclein and pS129 in PD subjects (Harapan et al., 2020). In fact, there are multiple problems of using α-synuclein pathology in mucosal biopsies from the gastrointestinal tract in the diagnosis of PD (Visanji et al., 2013; Chung et al., 2015). Further, the content of mucosal α-synuclein in the gastrointestinal tract in sporadic PD patients was claimed to vary with *SNCA* variants present in those subjects (Chung et al., 2019). Nevertheless, a recent systematic review and meta-analysis confirmed a strong association between gut α-synuclein and PD (Bu et al., 2019).

The accumulation of α-synuclein in neural structures within the submandibular gland in PD was demonstrated in multiple studies showing a good degree of consistency. Del Tredici et al. (2010) analyzed the deposition of α-synuclein immuno-histochemically in the submandibular gland and anatomically related sympathetic nerves and ganglion as well as the vagal nerve in autopsy study. The study included pathologically confirmed 9 PD patients, 19 controls, and some patients of MSA and incidental Lewy body disease. α-Synuclein immunoreactivity was observed in tissue samples in nearly all cases of PD, but none in the control or MSA subjects (Del Tredici et al., 2010). However, in a recent study of 59 cases of PD (early, moderate and advanced) and 21 healthy controls, biopsies from the submandibular gland showed immuno-reactivity to α-synuclein with a specific monoclonal antibody only in 56.1% cases but with 100% specificity (Chahine et al., 2020). Another study demonstrated that in the submandibular gland total α-synuclein (measured by a sensitive laser-based immuno-assay) in PD cases was similar to that in controls. However, the oligomeric form of α-synuclein (o-α-syn) content (measured by gel filtration followed by immuno-blotting) was higher in PD cases than in controls (Kang et al., 2016). This study further showed that total α-synuclein content in submandibular salivary gland tissue in PD cases varied with specific *SNCA* variants present in the patients. In a couple of studies, Beach and his group showed that immuno-reactive pS129 was present conspicuously in submandibular salivary gland tissue collected from autopsied persons in a high percentage of PD cases that were absent in controls (Beach et al., 2013, 2016). The studies considered only the immuno-reactive α-synuclein in nerve fibers within the salivary gland, and apart from PD cases, pS129 immunoreactivity was also present in a significant percentage of Lewy body dementia cases. Real-time quaking-induced conversion assay with the post-mortem submandibular salivary gland tissue (kept frozen or as paraffin-embedded tissue sections) reported a higher aggregation-seeding activity of pS129 in all cases of PD compared to control (Manne et al., 2020).

### α-Synuclein in Retina

**FIGURE 1:**
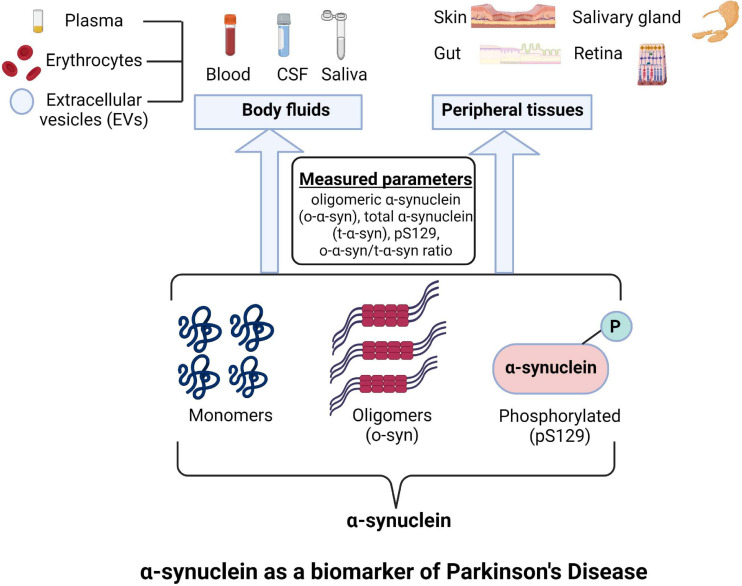
Different forms of α-Synuclein as a prospective biomarkers in Parkinson’s disease (PD). Image was created using BioRender.com.

Endogenous α-synuclein is present in all three different layers but predominantly in the inner plexiform layer in the retina (Surguchov et al., 2001; Beach et al., 2014). Visual problems and structural alterations in the retina in PD are being investigated intensively especially with techniques like optical coherence tomography (OCT), electroretinography (ERG), and visual evoked potentials (VEP) (Indrieri et al., 2020). The visual alterations in PD could be linked to α-synucleinopathy in the retina, which may also reflect the PD pathology in the brain (Veys et al., 2019; Indrieri et al., 2020). Many animal models of PD develop α-synucleinopathy in the retina (Price et al., 2016; Mammadova et al., 2019). From the point of view of the biomarker potential of retinal α-synuclein in PD, only limited information is available. Bodis-Wollner et al. (2014) reported the existence of α-synuclein aggregates in the retina of patients with PD and implicated their role in impaired vision and retinal morphology in the latter condition. Beach et al. (2014) also reported the presence of pS129 in the nerve fibers at the inner retinal surface in PD in contrast to controls. Likewise, in another study, pS129 immunoreactivity was reported in the retina in PD patients but not in controls; the deposits of pS129 appearing like LBs and LNs were observed in ganglion cells in perikarya as well as axonal and dendritic processes (Ortuño-Lizarán et al., 2018). This study mentioned that α-synuclein immunoreactivity in the retina was similar in control and PD subjects. On the other hand, another group used enucleated eyes at autopsy and observed α-synuclein immunoreactivity in the retina both in PD patients (67%) and controls (50%) without any indication of LBs and LNs in the patients (Ho et al., 2014). Figure 1 schematically summarizes the different forms of α-synuclein in biofluids and peripheral tissues studied to date as prospective biomarkers in PD.

## Conclusion and Future Direction

It is evident from the present discussion that though α-synuclein is a promising biomarker candidate, its specificity as a single diagnostic or prognostic biomarker of PD is far from established in any of the biological fluids or peripheral tissues. Some obvious reasons for this failure lie in the significant degree of inconsistencies among different studies, small sample sizes in predominantly cross-sectional studies, recruitment of patients with different degrees of disease severity and duration in different studies, and lack of participation of early PD patients in most studies. In addition, most studies compared PD with healthy controls or neurological controls but did not compare PD with other parkinsonian disorders or other kinds of movement disorders that may be confused with PD.

The technical issues of α-synuclein measurements in biological fluids are also very serious in the context of the variability of results in different studies. The chances of contamination of CSF with blood or that of plasma and serum with lysed red blood cells cannot be ignored. More serious could be the variations in immuno-assays, immuno-blotting methods, and various other purification procedures adopted by the different groups. α-Synuclein exists in monomeric and different types of oligomeric forms as well as in different post-translationally modified forms, and it is not known with what efficacy the antibody to one form of α-synuclein (supplied by the commercial manufacturers or produced in the lab by different groups) cross-reacts with other forms of α-synuclein in a particular assay. This could add serious errors to the measurement, making comparisons difficult among different studies. As already indicated, SNCA variants can result in different amounts of α-synuclein in different PD subjects, which might have also contributed to differences between the two studies. Further, most studies did not mention the storage of biological samples (time period and temperature of storage) before immunoassays were performed. Further, the effect of storage on the conversion of monomeric to different oligomeric forms or the stability of post-translationally modified forms of α-synuclein were also not analyzed. In other words, properly validated immunoassay methods are paramount in establishing the biomarker role of α-synuclein in PD. Likewise, for the peripheral tissue α-synuclein, the variations in immunohistochemical techniques and the lack of knowledge on different forms of α-synuclein present in the tissue in normal and pathological conditions and the limited number of commercially available antibodies are the major concerns. In most publications with tissue α-synuclein, the presence of immuno-reactivity to α-synuclein or pS129 was thought to indicate LB pathology without ascertaining the oligomeric state of α-synuclein present in the tissue.

Having discussed these limitations, we can say that measurements of o-α-syn, total α-synuclein, pS129 with or without total tau in CSF or red blood cell lysate or the saliva could be important in finally developing a good biomarker for PD. Future studies should focus more on red blood cell lysate and saliva in this regard. Likewise, the measurement of total α-synuclein in neuron-derived EVs may be equally promising in this context. For tissue α-synuclein and its oligomers, the skin biopsy studies, though limited in number, so far have provided consistent results. Further, a skin biopsy is a minimally invasive procedure that would be acceptable to PD patients. So the assessment of α-synuclein deposition in cutaneous nerve fibers in skin biopsy specimens in PD patients may emerge as a future diagnostic method. The same may be said for submandibular salivary gland α-synuclein in PD cases where the published results are very consistent, and thus larger studies should be conducted in the future with needle biopsy specimens from this gland. It may be equally important to use dyes like Thioflavin-S, which can bind to α-synuclein oligomers to give rise to fluorescent products that could be detectable under a fluorescence microscope. This can provide better information on the oligomeric state of α-synuclein in peripheral tissue. Intra-vitreal administration of Thioflavin S or a similar compound for visualization of fluorescent dye-bound α-synuclein aggregates in the retina, taking into consideration the toxicity angle, should be a future endeavor. Overall, it appears that establishing α-synuclein as a good biomarker for PD will require well-validated detection methods and larger clinical studies (cross-sectional and follow-up) with PD patients, patients of parkinsonian disorders, and non-parkinsonian movement disorders. The use of a combination of imaging biomarkers or other biochemical biomarkers along with α-synuclein for the diagnostic and prognostic evaluation of PD should also be explored more vigorously.

## Author Contributions

UG contributed to the conceptualization, writing, editing, and project management. SS and SoP contributed to the writing. SuP and BA contributed to the editing and supervision. RS contributed to the conceptualization, editing, and supervision. SC contributed to the conceptualization, writing, editing, supervision, and project management. All authors contributed to the article and approved the submitted version.

## Conflict of Interest

The authors declare that the research was conducted in the absence of any commercial or financial relationships that could be construed as a potential conflict of interest.
